# The Influence of Anhydrite on the Mechanical Performance of Calcium Sulfoaluminate Cement-Based Grouting Materials

**DOI:** 10.3390/ma18071547

**Published:** 2025-03-29

**Authors:** Lin Liao, Nathan Saye Teah, Zhiling Liao, Ruiqing Hao, Yubin Zhao, Wanwen Xue

**Affiliations:** Department of Underground Engineering, College of Mining Engineering, Taiyuan University of Technology, 79 Yingze West Street, Taiyuan 030024, China; nathanteah93@gmail.com (N.S.T.); lzlfight66@163.com (Z.L.); rqhao_tyut@163.com (R.H.); zhaoyubin1309@link.tyut.edu.cn (Y.Z.); xueww1112@163.com (W.X.)

**Keywords:** calcium sulfoaluminate cement, grouting material, anhydrite, quicklime, hydration mechanism

## Abstract

Cement-based grouting reinforcement technology is an essential method to enhance the mechanical performance of fractured rock depending on the type of grouting material used. To further understand the influence of anhydrite on the calcium sulfoaluminate cement-based grouting materials, this study investigates the sample of the grouting material with (calcium sulfoaluminate cement, anhydrite, and quicklime) under different ratios with a series of experiments including compressive strength, setting time, and slurry pH test. The macro and micro mechanical characteristics of different calcium sulfoaluminate cement grouting samples were revealed using X-ray diffraction (XRD) and scanning electron microscopy (SEM). The results indicate that anhydrite enriched the system with needle-like ettringite (AFt), and plume-shaped aluminum hydroxide (AH_3_), which contributed to the strength improvement. The optimal dosage of anhydrite-quicklime was 80:20 with a compressive strength of 9.5 MPa, 14.7 MPa, and 18.4 MPa at (1 d, 14 d, and 28 d), making up 38.5% of the total strength, and 80% independent of the quicklime dosage (14.2 MPa, 23.2 MPa, and 24.5 MPa), making up 52.5% of the total strength. Furthermore, X-ray diffraction and scanning electron microscopy results proved that anhydrite ratios are the main factor influencing grouting material reinforcement effectiveness and are more beneficial for improving the mechanical performance of calcium sulfoaluminate cement-based grouting material.

## 1. Introduction

The structural anchoring technique is an essential component of structural engineering that prevents and mitigates disasters. Anchoring techniques, as an important area of structural engineering, are used extensively in many construction projects, such as tunnel engineering, slope engineering, mining engineering, and foundation supporting due to their efficient and cost-effective benefits [1,2,3,4,5]. Meanwhile, as an essential element of anchorage structure, grouting technology plays a vital role in improving the mechanical properties of rock masses, self-stabilizing the surrounding rock’s capacity, reducing the likelihood of the surrounding rock mass instability and failure, and successfully preventing stress concentration in fracture zones [6].

In terms of cementitious material selection for anchoring grouting, PC and CSA cementitious are now used as the most common cementitious materials in anchoring grouting [7,8,9]. For over a century, PC hydraulic binders have been utilized as a grouting material in construction [10,11,12]. However, as a grouting material, it is prone to extended setting times, low early strength, and a smaller grouting range [13,14,15]. Additionally, it is important to recognize that PC manufacturing is not environmentally sustainable [16,17]. In the context of global warming and greenhouse gas emission reduction, it may result in a rise in the overall emissions of carbon dioxide (CO_2_) during the production process [18,19]. Therefore, to guarantee the best grouting performance, it is essential to enhance the extraordinary grouting material’s fluidity, setting time, expansion rate, and mechanical strength.

Calcium sulfoaluminate cement (CSA) is a hydraulic binder and an inorganic material that was developed in the 1970s and has been utilized and standardized in the construction and underground industry in China for more than a century [20,21]. Furthermore, CSA has positive effects on the environment. For instance, research shows that CSA cement can reduce carbon dioxide (CO_2_) emissions in China by roughly 35–48% [22,23]. It can also be marketed as an environmentally friendly alternative to PC because of its quick setting time, early high strength, and low CO_2_ emissions. However, because CSA cement has properties like low clinker sintering temperature, low limestone consumption, and strong chemical corrosion resistance, it can be considered a good grouting cementitious material and an alternative method of reducing greenhouse gas emissions in the context of global warming [24,25,26], and its addition to anhydrite can result in a larger concentration of ye’elimite phase minerals [27,28].

In this study, the CSA cement is mainly composed of ye’elimite (C_4_A_3_$\bar{S}$), and belite (C_2_S). In addition, it also contains some trace minerals, such as Iron (III) oxide (Fe_2_O_3_), (graphite fluoride) C_2_F, as shown in Table 1. In the CSA cementitious material system, ettringite (C_6_A$\bar{S}$_3_H_32_) production is mostly dependent on ye’elimite (C_4_A_3_$\bar{S})\;$. There are two ways that the hydration process of ye’elimite (C_4_A_3_$\bar{S}$) plays a crucial role in the ettringite formation. First, anhydrite (C$\bar{S}$) typically occurs in the hydration of ye’elimite (C_4_A_3_$\bar{S}$) to produce ettringite (C_6_A$\bar{S}$_3_H_32_) and hydroxide aluminum (AH_3_) via reaction (1). This results in high early strength and quick hardening of the CSA cement paste [29]. Second, if there is not enough anhydrite (C$\bar{S}$), ye’elimite (C_4_A_3_$\bar{S})\;$ may hydrate to produce mono-sulfoaluminate (C_4_A$\bar{S}$H_12_) via reaction (2). Because of the quick reactivity of ye’elimite (C_4_A_3_$\bar{S})\;$and the expansion of the cement structure as ettringite (C_6_A$\bar{S}$_3_H_32_) forms (Equation (2)), CSA cement causes rapid coagulation and expansion characteristics [30]. This characteristic makes it useful for precast products, self-repairing concrete, and quick repairs.

(1) $$C_{4}A_{3}\bar{S}+2C\bar{S}H_{2}+38H⟶C_{3}A\cdot 3C\bar{S}\cdot 32H+2AH_{3}$$

(2) $$C_{4}A_{3}\bar{S}+18H⟶C_{3}A\cdot C\bar{S}\cdot 12H+2AH_{3}$$

Although CSA cement has better material performance, its limited raw ingredients, high cost, and poor alkalinity limit its engineering use [31,32]. To improve the engineering performance of CSA cement, researchers have explored possibilities for using mixed materials and supplemental cementitious materials (SCMs). Research has indicated that incorporating fly ash, limestone powder, calcite, and anhydrite can enhance the engineering performance of CSA cement [33,34]. Currently, the usage of limestone in cement is receiving a lot of attention. In the case of CSA cement paste mixed with limestone powder, Pelletiter et al. [35] utilized limestone powder in CSA cement. It speeds up the early hydration of CSA cement because of its filler effect, thereby promoting the early age of heat evolution in CSA cement. Furthermore, the limestone powder can fill in the pores of cementitious materials, thus reducing the porosity of cement-based materials [36], and improving the strength, permeability, carbonation resistance, and corrosion resistance of reinforced concrete [37,38]. Xie et al. [39] utilized calcium carbonate, and limestone powder in PC-CSA clinker. The mixture reacted well, thereby improving the ettringite. This is because the generation of calcium carboaluminate can stabilize ettringite causing low porosity at early ages. The calcite in CSA cement can react with mono-sulphoaluminate (AFm) to form ettringite and mono-carbo aluminate, as shown in Equations (3) and (4).

(3) $$3C4A3\bar{S}+2C\bar{C}+72H⟶2C4A\bar{C}H11+C3A\cdots 3C4\bar{S}\cdots 32H+6AH3$$

(4) $$6C4A3\bar{S}+C\bar{C}+135H⟶2C4A\bar{C}\;\;0.5H11+2C3A\cdots 3C4\bar{S}\cdots 32H+6AH3+14AH3$$

Additionally, in the case of anhydrite, Pelletier et al. [40] utilized anhydrite in a ternary system with PC–CSA clinker; the anhydrite promotes early hydration and allows proper hydration mechanism and reaction formation (Equation (5)). Anhydrite is a natural product, and its environmental impact improves binder hydration. Moreover, following the stoichiometry reaction of Equation (5), the hydration characteristic of the quantity of anhydrite represents around half of the ye’elimite weight (anhydrite weight/ye’elimite weight = 0.45), which had significant rapid hardening, early strength, and micro expansion characteristics as a CSA-based grouting material. In ternary blends of CSA, anhydrite, and limestone, anhydrite is more reactive with CSA clinker than limestone [41]. The production of ettringite and mono-carbonate rises with increasing anhydrite content, whereas mono-sulfoaluminate content falls.

Moreover, early hydration is accelerated as the molar ratio (M) of anhydrite to ye’elimite rises. This happens because of the reactive bonding anhydrite has with CSA cement. This bonding creates a relationship between ye’elimite and calcium sulfate, which makes them contribute immensely to the production of ettringite secondary. According to Zhou et al. [42], when excess anhydrite (C$\bar{S}$) is incorporated into a CSA–OP cement grouting material mix, rapid hydration of ye’elimite (C_4_A_3_) will occur, leading to the formation of ettringite (AFt) crystal and aluminum hydroxide (AH_3_). Adding CSA and Portland cement will boost strength and durability. However, incorporating the proper dosage of anhydrite (C$\bar{S}$) will make the cement mortar more durable and stable enough to sustain pressure and stress during the construction phase.

(5) $$C_{4}A_{3}\bar{S}+2C\bar{S}+38H⟶C_{6}A{\bar{S}}_{3}H_{32}+2AH_{3}$$

The above research findings have significantly contributed to the extensive and comprehensive application of calcium sulphoaluminate (CSA) cement as a cement-based grouting material. However, there is limited research on how the mechanical qualities and performance of CSA cement-based grouting materials are influenced by anhydrite. To meet the practical application requirements, this research explores methods to enhance the early and later age compressive strength of the grouting materials by incorporating anhydrite into the cement-based CSA, thereby creating a novel cement-based grouting material. Additionally, quicklime and anhydrite are added to influence the hydration reactions of the grouting materials, and their impact on the compressive strength, expansion, pH, and setting time is studied. The hydration process and the micro-level effects of anhydrite on the novel CSA cement-based grouting materials are investigated using X-ray diffraction (XRD) and scanning electron microscopy (SEM). The results of this research can offer valuable insights into the application of CSA-anhydrite-based grouting material.

## 2. Materials and Methods

### 2.1. Materials

The cement used in this study is a commercial CSA 42.5 cement produced by Shanxi Zhongbao Construction and Building Materials Co. of China, Ltd. (Taiyuan, China). It has a specific surface area of 450 m^2^/kg and a measured density of 2.89 g/cm^3^, according to the factory performance data. The CSA cement mineral composition was characterized through a Rietveld refinement X-ray diffraction analyzer (XRD) version 5.3, as shown in Table 1, and chemical components through an XRD analyzer (Rietveld refinement), as shown in Table 2. The anhydrite used in this study is off-white. It was purchased from Changzhi Zhongbao Building Material Co. of China, Ltd., Taiyuan, China, and has a density of 2.85 g/cm^3^. It was formed from calcium sulfate (CS) in an aqueous solution that was deposited as gypsum crystals. Its chemical composition was determined through the Malvern Panalytical X-ray fluorescence (XRF), as shown in Table 3. In the case of CSA cement, when incorporated with anhydrite or reacted with sulfuric anhydrite (SO_3_), the anhydrite serves as a regulator to control tricalcium aluminate (C_3_A) hydration rate and produce more ettringite. The quicklime in this study appears white, with a density of 3.1 g/cm^3^, has an effective calcium oxide content of more than 90%, and is supplied by Xilong Scientific Co., Ltd., Guangzhou, China. The quicklime easily reacts with water to form calcium hydroxide (Ca(OH)_2_).

### 2.2. Mix Proportions

This research intends to study the influence of anhydrite on the mechanical performance of CSA cement-based grouting materials. To achieve that, this research experiment compares the influence of anhydrite in two distant forms. First, based on the quality of the CSA clinker, the sample of CSA does not change and the anhydrite content is incorporated alone with a water-cement ratio of 0.5 to see its influence on the CSA cement grouting material. At this stage of the experiment, the content of anhydrite began from low to high (0–100%) and did not necessarily depend on the content of CSA cement to summarize the composition, as shown in Table 4.

Second, anhydrite is incorporated with quicklime under the same water–cement ratio to determine how it will react to the CSA cement as a grouting material. The ratio of both materials is incorporated at equilibrium with the mass ratio of CSA cement: (anhydrite + quicklime) is 1:1, in which the ratio of anhydrite and quicklime is changed from 1:1 to 8.5:1.5, as shown in Table 5. During these two forms of experimental procedures on anhydrite influence, the calcium sulfoaluminate cement-based grouting materials mixed mixture does not secrete water and sets the grouting material rapidly. As the test ingredients are displayed in Table 4 and Table 5, the ideal ratio of the calcium sulfoaluminate cement-based grouting materials was not simply selected; rather, it was attained through extensive experimentation and natural maintenance conditions to the designated age. The quality of the anhydrite and the water-to-cement ratio were employed in the slurries to satisfy the flow of the grouting material and study the basis of the experiment.

### 2.3. Test Methods

#### 2.3.1. Compressive Strength Test

**Figure 1:**
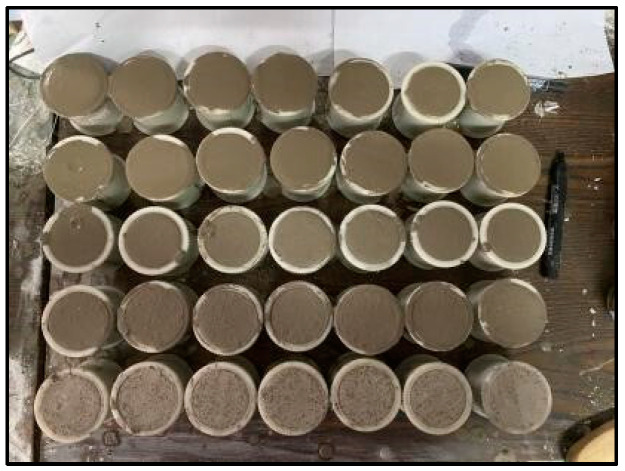
Forming mold.

**Figure 2:**
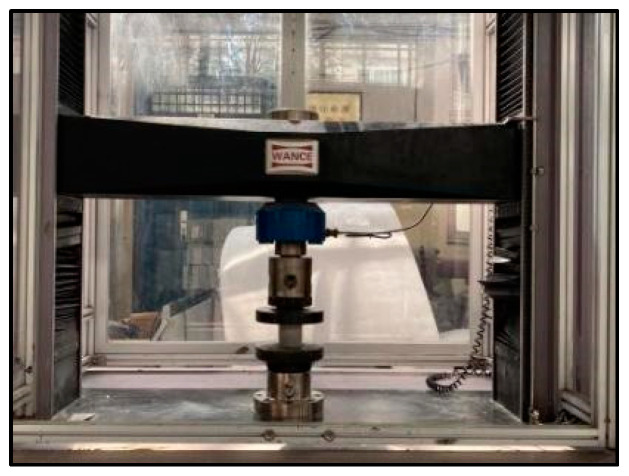
Compressive strength test.

According to the shape and tolerances of test specimens standard for testing the method of mechanical properties of ordinary concrete GB/T 50081-2002 [43], the specimen compressive strength was measured. As shown in Figure 1, the blended paste slurry was poured into a 50 mm × 50 mm × 50 mm mold and was prepared according to the mix proportion, as shown in Section 2.2. The paste test block compressive strength was measured for 1 d, 14 d, and 28 d using the ETM205D-TS compression testing equipment, at a loading rate of 0.3 MPa/s. According to the experiment proportion, the sample per series for each ratio is 1:4 (one proportion ratio–four test series). The average value of four replicates was taken as the experiment value, as shown in Figure 2.

#### 2.3.2. pH Test

The pH of the slurry was tested using a PHS-3C precision pH tester produced by the Nanjing T-Bota Scietech Instruments & Equipment Co., Ltd., Nanjing, China. The slurry was stirred for 3 min to make it homogeneous. The calibrated pH meter test pen was inserted into the slurry, and readings were taken when the pH had stabilized.

#### 2.3.3. Setting Time Test

After the blending of the slurry, it was poured into a Vicat apparatus mold. The vicat apparatus was used to measure the initial and final setting times of the blended cement paste by complying with ASTM C191 [44]. The penetration of the Vicat needle determined the initial and final setting times of the blending cement paste at 26 ± 4 mm and 0.5 ± 0.5 mm, respectively.

#### 2.3.4. Expansion Test

**Figure 3:**
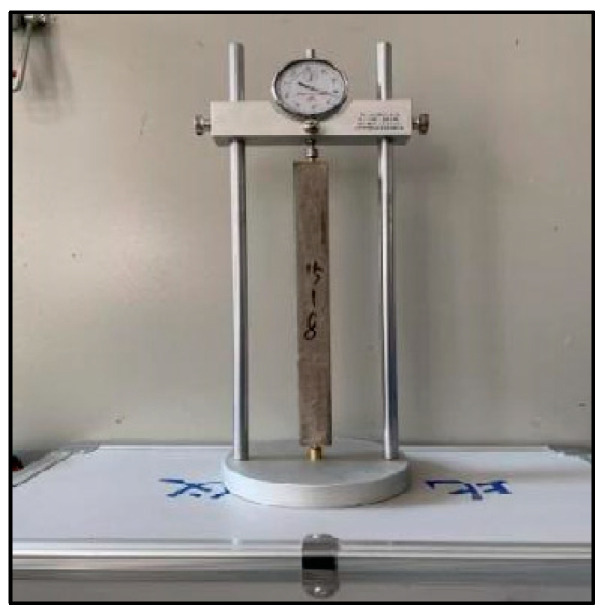
Expansion-shrinkage test device.

**Figure 4:**
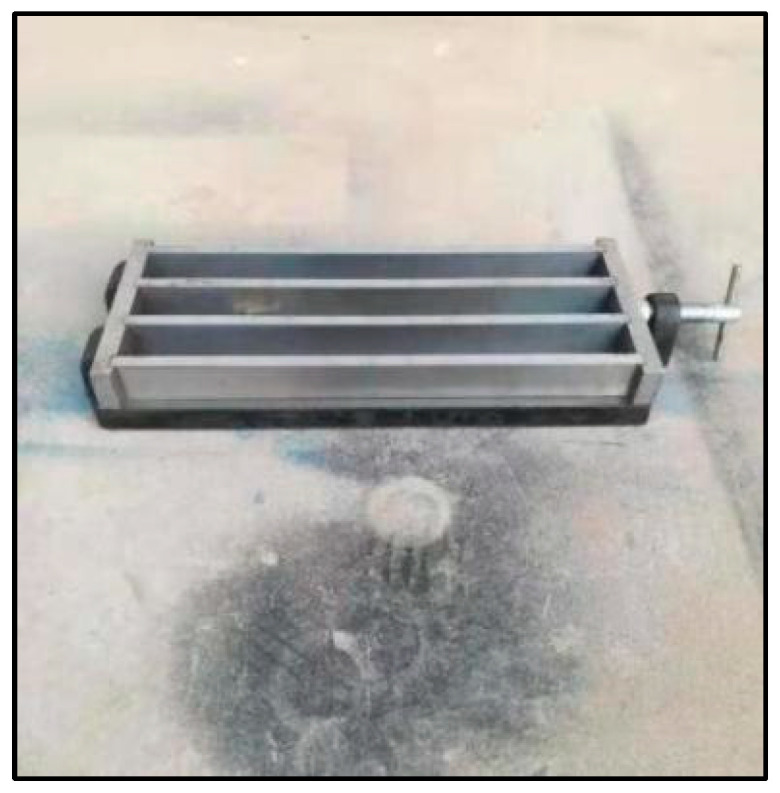
Triplex steel mold.

The paste specimen expansion stability was studied using thermomechanical methods on solid material [45]. The slurry paste was blended for 3-mins and poured into a mold of 50 mm × 50 mm × 50 mm. After demolding, the initial lengths (L_0_) of the specimens were measured as shown in Figure 3, then the specimens were cured in water at 80 °C for 24 h. Starting from the day on which the initial lengths were measured, the changes in the specimens were observed at 1 d 14 d, and 28 d, and the corresponding lengths (L_1_) were measured using the triplex steel film of 25 mm × 25 mm × 280 mm as shown in Figure 4. The formula for the calculation of the expansion rates (E_X_) of the specimens is given in Equation (6), and the average value of the expansion rates of the four specimens was regarded as the result for each group of specimens.

(6) $$E_{x}=\frac{L_{1}-L_{0}}{{L_{0}-(∆}_{1}-{∆}_{2})}\times 100$$

where E_X_ is the expansion rate of a specimen at the observed day (%); L_1_ is the length of a specimen at the observed day (mm); L_0_ is the initial length of a specimen (mm); and Δ_1_ and Δ_2_ are the lengths of the measuring heads at the left and right ends of the specimen (mm).

#### 2.3.5. X-Ray Diffraction (XRD)

The hydrated samples were soaked in anhydrous ethanol for 3 d to stop the hydration. The hydrated specimen was dried in a vacuum drying oven. The temperature and the vacuum degree were 40 °C and 0.08 Mpa, respectively. After heating, the hydrated specimens were ground by hand into a powder below 0.025 mm and passed through a 200-mesh sieve. An X-ray diffraction analyzer (Rietveld refinement) was used to determine the mineralogical composition of the hydrated specimen. The test range was 5–80° at a scanning rate of 10°/min.

#### 2.3.6. Scanning Electron Microscopy (SEM)

The samples were taken from the same hydrated sample that was immersed in anhydrous ethanol for 3 d. The hydrated samples were ground flat to a size of less than 1 cm. Then, the grounded specimen was examined by scanning electron microscopy (ZEISS Gemini SEM 300 produced in Oberkochen, Germany) using a secondary electron image and energy-dispersive X-ray (Rietveld refinement) analysis.

## 3. Results and Discussion

### 3.1. Compressive Strength

**Figure 5:**
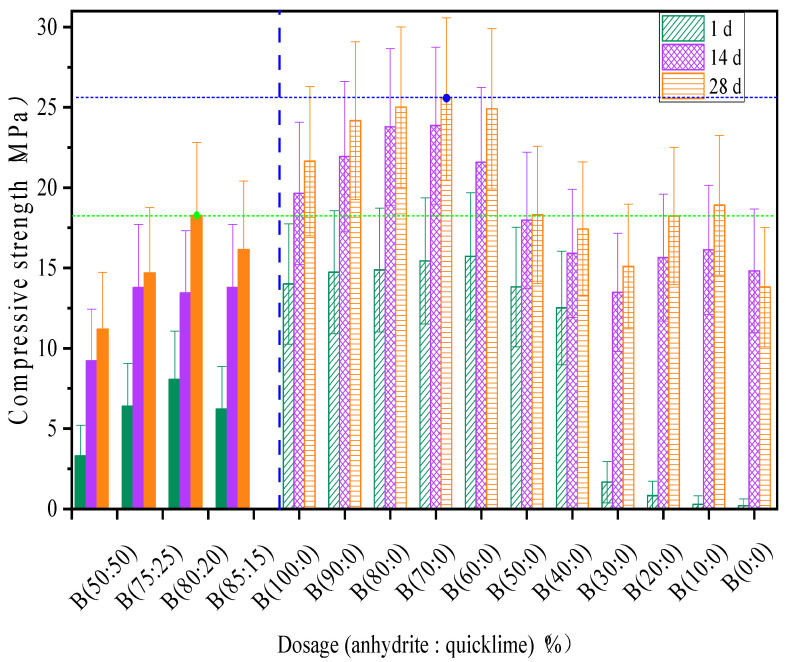
Effect of anhydrite–quicklime dosage on compressive strength of slurry B at different ages.

Figure 5 shows that when only CSA cement was used, the overall trend of the strength change curve fluctuated. The 1 d, 14 d, and 28 d strengths were 2.52 MPa, 122 MPa., and 10.4 MPa, respectively. As ≤30% of anhydrite dosage was incorporated, there was little increase in the early strength. The 1 d strength was 2.2 MPa at 10%, 2.72 MPa at 20%, and 3.2 MPa at 30%, respectively. However, as the curing age increases, the strength increases with a little fluctuation. The 14 d strengths are 15.2 MPa, 15.8 MPa, and 14.8 MPa, while the 28 d strengths are 19.4 MPa, 19 MPa, and 15 MPa, respectively. The cause of the decrease in the early strength was the fact that anhydrite could not participate in the early hydration because of its low dosage incorporation. However, as the curing age increases, the calcium silicate (CS) and sulfuric anhydrite (SO_3_) react in the hydration reaction and produce more ettringite (AFt) to improve the strength. Following the increase in the anhydrite ratio to ≤60%, the strengths of the 1 d, 14 d, and 28 d showed a rapid increase at an early age. The 1 d strengths are 11.4 MPa at 40%, 12.05 MPa at 50%, and 15.01 MPa at 60%. The 14 d strengths are 14.7 MPa at 40%, 15.3 MPa at 50%, and 18.2 MPa at 60%, while the 28 d strengths are 15.2 MPa at 40%, 15.7 MPa at 50%, and 23.5 MPa at 60%, respectively. The cause of the increase in the early strength was the fact that anhydrite participated in the early hydration and improved the strength. As anhydrite dosage increases to ≤100%, 80% dosage is the most appropriate. The trend curve shows a more significant rise at all ages. The 1 d, 14 d, and 28 d strengths are 14.22 MPa, 23.2 MPa, and 24.52 MPa. They contribute to 10.2%, 32.3%, and 42.5% of the overall strength of anhydrite alone, respectively. At 90% dosage, the strength curve increases continuously. The 1 d, 14 d, and 28 d strengths are 14.85 MPa, 21.5 MPa, and 23.7 MPa, respectively. At 100% dosage, the strengths of the 1 d, 14 d, and 28 d, are 14.3 MPa, 18.22 MPa, and 20.52 MPa. This shows that the impact of anhydrite on the strength of the CSA grouting material depends on the dosage.

Following the incorporation of an anhydrite–quicklime ratio, the mixture with only CSA cement was used, and the overall trend of the strength change curve was the same as earlier. As anhydrite–quicklime (50:50) dosage was incorporated, the compressive strength change curve exhibited different trends. The 1 d, 14 d, and 28 d strengths are 1.7 MPa, 9.7 MPa, and 12.5 MPa which are 5% and 11.5% more than the compressive strength in the slurry with CSA alone at the early and final age. The incorporation of anhydrite-quicklime regulates the early hydration of the CSA cement that is facilitated by tricalcium aluminate (C_3_A).

As the mixture ratio of anhydrite–quicklime increased to a 75:25 ratio, the strengths of the 1 d, 14 d, and 28 d were 7.5 MPa, 13.5 MPa, and 14.8 MPa. The strength was almost two times better than the 50:50 ratio at all ages. This indicates that when the anhydrite–quicklime ratio is at equilibrium, it delays the hydration which could lead to a decrease in the early strength. Following the change in the ratio of anhydrite–quicklime, the 80:20 ratio was the best. It has the highest compressive strength at all ages, reaching 9.5 MPa at 1 d, 14.7 MPa at 14 d, and 18.4 MPa at 28 d, which increases the strength to 10.2%, 20.3%, and 38.5%, respectively. At an 85:15 ratio, there was a little decrease in the early strength. The 1 d and 14 d strength curves exhibited the same trend as the 75:25 ratio. This decrease in strength was caused by the pores in the specimen and the slow participation of anhydrite in the hydration reaction. As the 85:25 ratio age increases to 28 d, the strength increases to 15.2 MPa. This shows that anhydrite has a great impact on CSAGM strength at both early and later ages when it reacts with CSA cement, and the decrease in quicklime dosage does not affect the impact on the specimen strength.

### 3.2. pH and Setting Time

**Figure 6:**
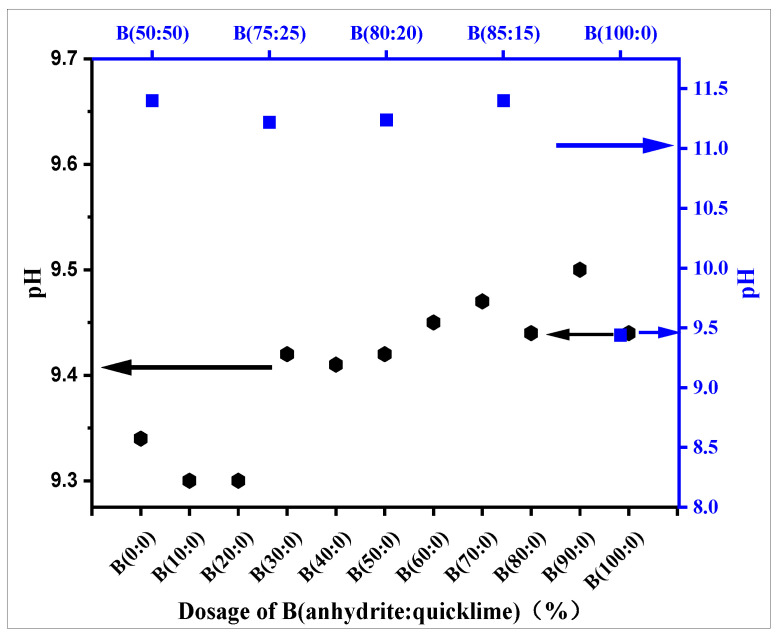
The pH of different anhydrite–quicklime admixtures on slurry B.

**Figure 7:**
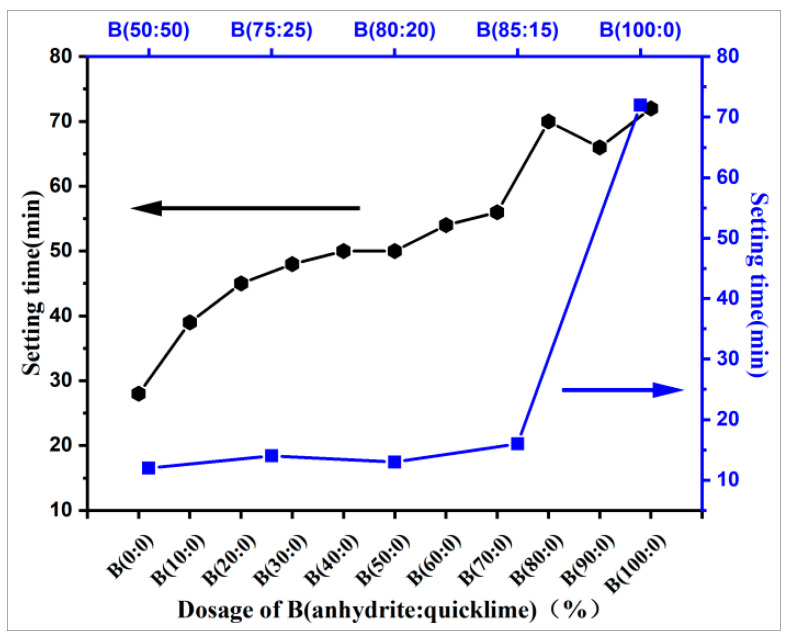
Slurry setting time at different anhydrite–quicklime dosages.

The length of the setting time is the basis of ensuring excellent flow. In addition to the use of admixture adjustment, the influence of raw materials is also very important. The slurry pH ensures the important factor of setting time, so it is necessary to study the pH and setting time for different ratios of the raw materials. Figure 6 and Figure 7 show the scatter diagrams of the slurry pH and the change curve of the setting time, respectively, as the CSAGM slurry increases with different dosages of anhydrite admixture.

Figure 6 shows that in the system of only CSA clinker and anhydrite, the different amounts of anhydrite admixture have little effect on the pH value of the CSAGM slurry, and the pH value varies between 9.30 and 9.50, with no large fluctuation or change pattern. These results, combined with the strength testing results, show that the solidification strength of the slurry with a low pH can also be high. When quicklime is added, the slurry’s pH value increases to approximately 11.3. The reason for this is that the quicklime dissolves in water and releases a large amount of OH-, which increases the alkalinity of the system, indirectly proving that the role of quicklime in the slurry material is mainly to increase the alkalinity of the system, thus speeding up the coagulation and promoting the hydration process [46]. This indicates that, as the relative proportion of quicklime increases, the setting time decreases rapidly, and as it becomes relatively large, it absorbs a large proportion of water because it raises the pH value of the slurry.

Following the increase in the proportion of anhydrite–quicklime admixture in slurries A and B, the setting time gradually increases and adheres to the surface of unreacted CSA clinker, thus extending the setting time and changing the overall linear pattern [47], as shown in Figure 7. In the case of CSA clinker (Slurry A) only, anhydrite admixture significantly prolongs the setting time of the clinker. This is because cement has high solubility and reactivity compared with admixture and the setting time of cement depends partly on the solubility of the cement material. In addition, this indicates that, as the amount of anhydrite increases, the hydration reaction is hindered.

### 3.3. Expansion Properties

**Figure 8:**
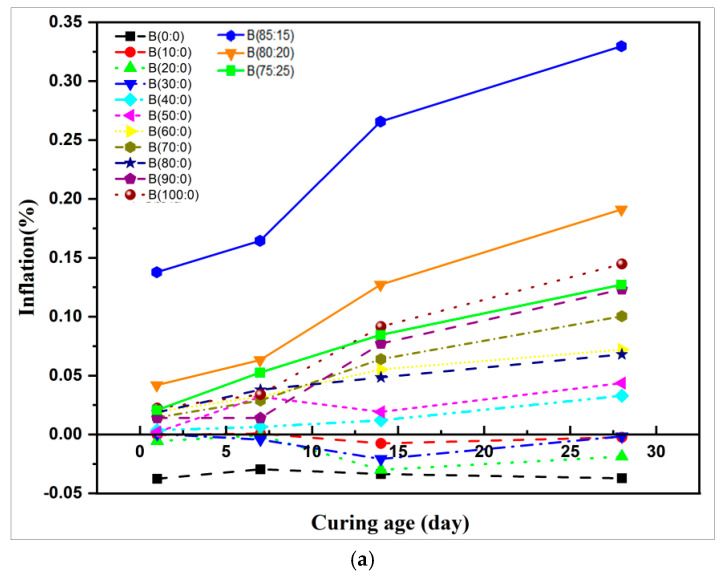

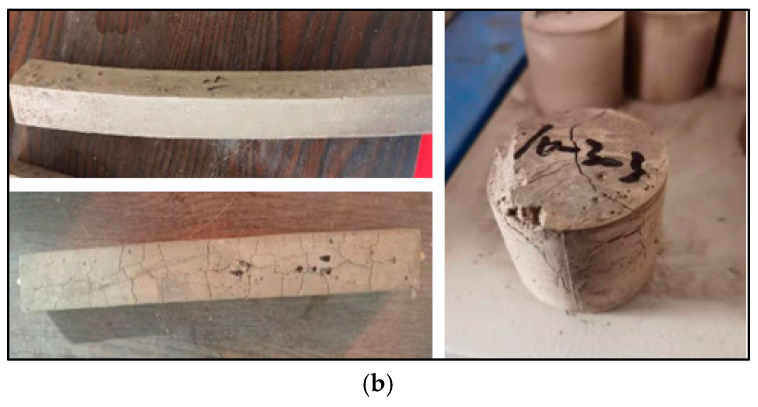
(**a**) Variation curve of the expansion rate at different anhydrite–quicklime dosages. (**b**) Sample damaged by expansion and cracking.

Appropriate expansion can improve the practical performance of the grouting material. However, if the expansion rate is too high, it will lead to the destruction of the material, so it is necessary to consider how to achieve an appropriate expansion rate as well as a good compressive strength. Figure 8a,b shows the effect of the anhydrite–quicklime admixture on the expansion rate of the specimen.

As shown in Figure 8a, when the slurry material is mixed with CSA cement (slurry A) and anhydrite alone, the shrinkage of the test block is obvious. Following the increase in the anhydrite–quicklime ratio of slurry B material to 50:50, the expansion and cracking damage could not be measured for the specimens at the age of 1 d (Figure 8b). The reason for this is that the increase in the anhydrite–quicklime dose is at an equilibrium ratio. Incorporating too much quicklime can affect the early and later age expansion performance of the specimen, which can cause the specimen hydration and strength growth to be slow, with cracking occurring on the specimen as a result of the system not effectively controlling the expansion performance, as shown in Figure 5 The change in expansion properties after the addition of quicklime is caused by the calcium alumina product on the one hand and the expansion of quicklime on the other (Figure 8b). This indicates that, as the quicklime content increases, the expansion rate decreases [48].

When the incorporation of anhydrite is less than 40%, the slurry material exhibits a certain degree of shrinkage at all ages, as shown in Figure 8b. The volumetric unsetting mechanism of traditional cement grouting material is observed in the fluid phase, due to the long setting time. This causes cement slurry to delaminate the water separation under the action of gravity of its particles, thus decreasing the amount of water absorbed. During the delamination process of the early hardening phase, the large rapid hydration reaction dissolves a large amount of mixing water, resulting in insufficient hydration and material shrinkage deformation. In addition, the low-anhydrite admixture appears to form alumina, ferric oxide (AFm) from ettringite (AFt) conversion, which causes the volume of the cured specimen to shrink.

When the anhydrite admixture exceeds 40%, the expansion rate gradually increases at all ages because of the increase in calcium alumina generated with cumulative expansion. The addition of anhydrite significantly increased the AFt content, but the expansion effect is related not only to the ettringite (AFt) content but also to the setting time of the cement. The analysis of the setting time of the specimens (Figure 7) showed that the final setting time was prolonged after the addition of anhydrite, and the formation of ettringite (AFt) probably occurred before the hardening of the paste, resulting in the consumption of expansion energy in plastic deformation. When the anhydrite admixture continued to increase, in addition to the expansion of AFt, the remaining anhydrite also expanded, but the amount of expansion was smaller compared to that when quicklime was added [49].

This indicates that different morphologies of ettringite (AFt) can produce different expansion effects. This result is in agreement with Bizzozero et al.’s research [47], which proposed that when the anhydrite admixture ratio is larger, it follows the threshold effect of expansion. This indicates that ettringite’s (AFt) capacity to expand and enter the system of interconnected pores is linked to its supersaturation. It can only apply pressure in a small number of isolated pores below the critical sulfate threshold. Once the critical sulfate threshold is reached, the pores will enlarge, increasing the total volume pressure produced by calcium alumina and causing expansion.

### 3.4. XRD Material Phase Hydration of the CSAGM

**Figure 9:**
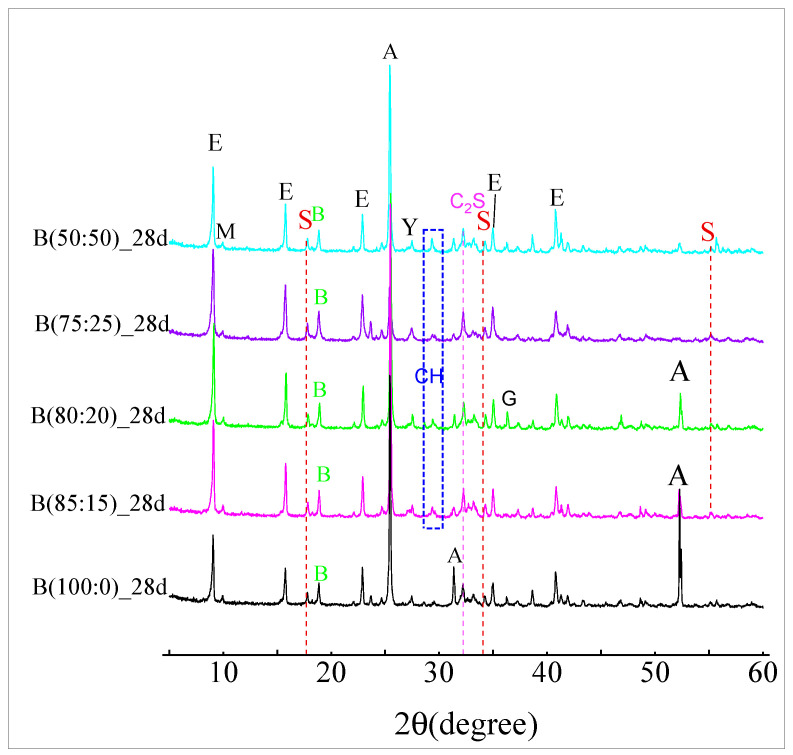
XRD at 28 d of age at different anhydrite-quicklime dosages (E: ettringite, Y: ye’elimite, A: anhydrite, M: alumina ferric-oxide, B: bayerite, G: gypsum, S: strätlingite).

Figure 9 shows the XRD of the CSAGM with different anhydrite–quicklime ratios at the age of 28 d. The decrease in the specimen strength of the grouting material with the increase in anhydrite admixture is due to the excess anhydrite generating a low-crystalline-phase gel similar to the coating stabilized on the surface of hydrated particles, which promoted calcium hydroxide (CH) and Sulfate (S) in the early phase, and inhibits the formation of ettringite (AFt) in the later phase. As the second product of anhydrous calcium sulfoaluminate hydration, aluminum hydroxide (AH_3_) is visible in form and co-exists as Al(OH)_3_ (bayerite and gibbsite). XRD hydration peaks of dicalcium silicate (C_2_S) were detected in the XRD pattern 32.026°, but the peak variation is not obvious, indicating that the anhydrite–quicklime admixture has no significant effect on the hydration of dicalcium silicate (C_2_S) in sulfoaluminate cement-based grouting materials.

In addition, the trace amounts of the hydration products katoite (C_2_ASH_8_) and calcium hydroxide (Ca(OH)_2_) (Equation (1)) were detected when quicklime was involved in the reaction due to the dissolution of quicklime into calcium hydroxide(Ca(OH)_2_), which inhibited the hydration of dicalcium silicate (C_2_S) [Equation (2)] from forming calcium silicate hydrate (C-S-H), while dicalcium silicate (C_2_S) reacted with aluminum hydroxide (AH_3_) to form strätlingite (C_2_ASH_8_). This explains why there are not any hydration products visible in the XRD diagram behind the weak calcium silicate hydrate (C-S-H) filling in the calcium alumina structure, as seen in Figure 6 and Equations (7) and (8).

C_2_S + 2H_2_O → C-S-H + Ca(OH)_2_ (7)

C_2_S + AH_3_∙5H_3_O-H + C_2_ ASH_8_ (8)

**Figure 10:**
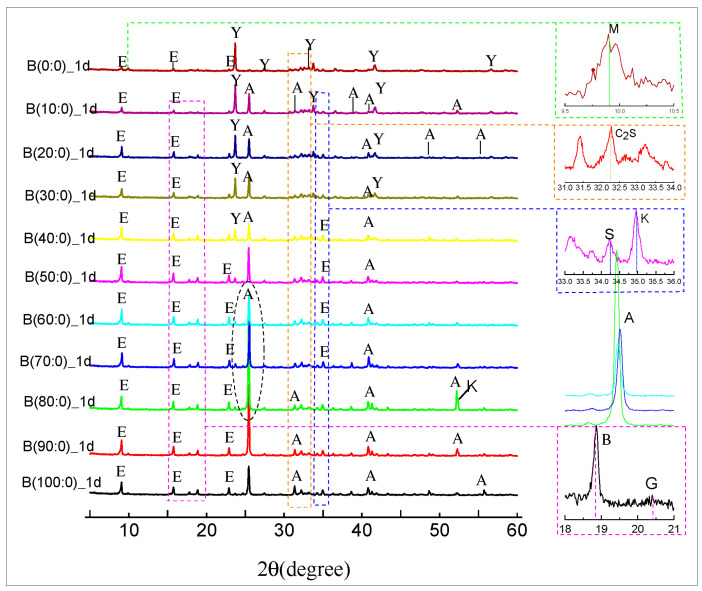
XRD at 1 d of age at different anhydrite dosages (E: ettringite, Y: ye’elimite, A: anhydrite, K: katoite, M: alumina ferric-oxide, B: bayerite, C: tricalcium aluminate, S: strätlingite, G: gypsum).

**Figure 11:**
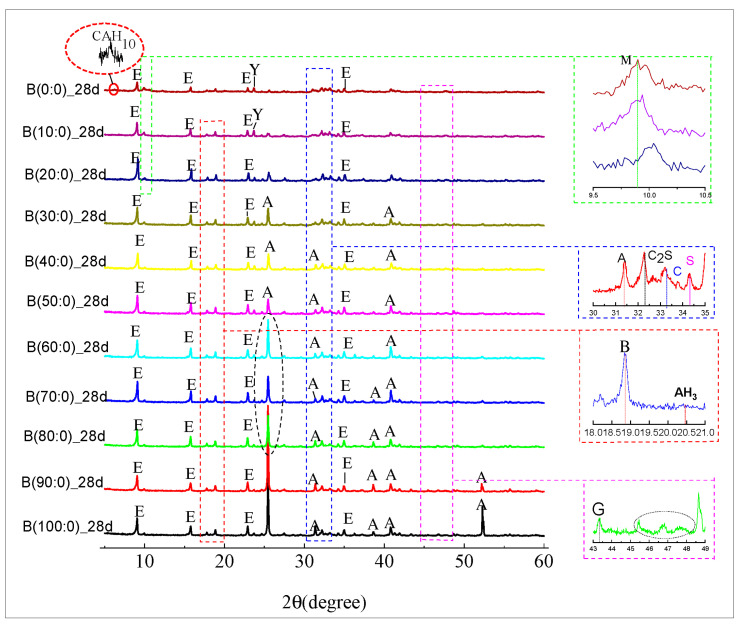
XRD at 28 d of age at different anhydrite dosages (E: ettringite, Y: ye’elimite, A: anhydrite, M: alumina ferric-oxide, B: bayerite, C: tricalcium aluminate G: gypsum, S: stratlingite).

**Figure 12:**
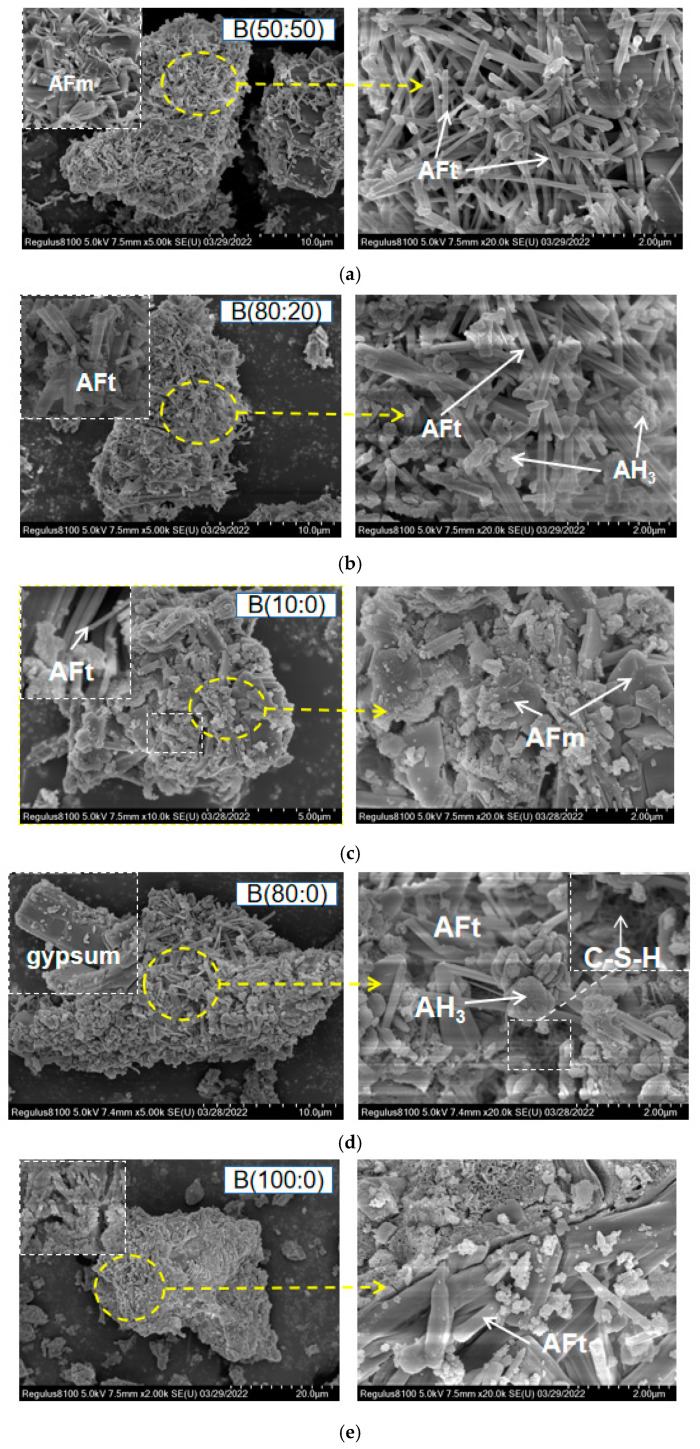
SEM of 28 d with different anhydrite dosage ((**a**): B(50:50); (**b**): B(80:20); (**c**): B(10:0); (**d**): B(80:20); (**e**): B(100:0)).

Figure 10 and Figure 11 show the XRD spectra of the hydration products of CSA clinker with different dosages of anhydrite admixtures at 1 d and 28 d of curing age. When anhydrite was not added, the formation of ettringite (AFt) was detected in the XRD of 1 d hydration of CSA clinker accompanied by the formation of AFm (Figure 11), but the reaction was not complete, and there was remaining CSA clinker in the system. At 28 d, almost no CSA clinker was monitored (Figure 12), the main hydration product was, ettringite (AFt) and the hydration reaction was complete.

As shown in Figure 10, AFm was detected at 28 d of reaction when 10 to 30 wt.% anhydrite was incorporated, indicating that the hydration continued with an increase in age. AFt was decomposed and transformed to alumina ferric-oxide (AFm) due to the lack of anhydrite incorporation or insufficient anhydrite incorporation. Following the increase in anhydrite ratio to 80% or more, the early phase was slow; the main hydration product was gypsum (G) and no longer CSA clinker at 1 d, indicating that anhydrite promoted the hydration reaction, and after the CSA clinker was completely reacted, there was also a slight increase in the peak intensity of ettringite (AFt) at the later stage, as seen in Figure 10.

Figure 10 shows that no ettringite (AFt) was found in the early phase of hydration when sulfoaluminate cement was used alone. However, the sulfoaluminate adsorption promoted the formation of calcium aluminate decahydrate (CAH_10_). The generation of strätlingite (Ca_3_A_l2_(OH)_12_) in the hydration product was detected at 35.053° after mixing with anhydrite, which has a significant effect on the strength improvement. This is because the generation of strätlingite (Ca_3_A_l2_(OH)_12_) is related to the amount of anhydrite. When the hydration of CSA clinker has been mostly completed, it is accompanied by the generation of strätlingite and aluminum hydroxide (AH_3_) because the remaining anhydrite and the straitlingite (Ca_3_A_l2_(OH)_12_) do not continue to hydrate into ettringite (AFt) [50].

The calcium silicate hydrate (C-S-H) gel was not detected (Figure 10 and Figure 11) in the XRD pattern during the whole reaction process because it is an amorphous substance and could not be detected by XRD. In addition, because of the high concentration of anhydrite in the hydrated environment, which induces the transition from alumina ferric-oxide (AFm), dicalcium silicate (C_2_S) to ettringite (AFt), the excess anhydrite will consume calcium hydroxide (Ca(OH)_2_) by reacting with calcium hydroxide (Ca(OH)_2_) and aluminum hydroxide (AH_3_). No diffraction peak of calcium hydroxide (Ca(OH)_2_) was detected in the XRD when anhydrite was mixed alone. Calcium hydroxide (Ca(OH)_2_) was detected (Figure 9) when quicklime was mixed in because the hydrated calcium hydroxide of quicklime was hardly involved in the hydration reaction. Tricalcium aluminate (3CaO∙A_l2_O_3_) and the sulfoaluminate type of hydrates were detected by XRD. Following the increase in anhydrite ratio and hydration process, aluminum hydroxide (AH_3_) gel is another hydration product that contributes to the strength after AFt to a certain extent, which is also verified by the highest production of aluminous gum at an 80% anhydrite dosage. Combined with their respective swelling rates at 28 d (Figure 8), excessive anhydrite dosage leads to expansion and thus strength reduction (Figure 5). The presence of gypsum can be significantly detected when the anhydrite dosage is 80% (Figure 11). The amount of dicalcium silicate (C_2_S) (Figure 10 and Figure 11) does not vary much, indicating that the anhydrite admixture has less effect on the degree of hydration of dicalcium silicate (C_2_S) in CSAGM.

### 3.5. SEM Material Phase Microstructure of the CSAGM

To study the effects of different anhydrite–quicklime admixture proportions on the microstructure of CSAGM, the morphology of the specimens at a specific age (28 days) was examined using SEM, and the representative micrographs were selected (Figure 12). The selected SEM images of different dosages of CSAGM slurry B are represented with B(50:50), B(80:20), B(10: 0), B(80: 0), and B(100:0). These were tested, and the main conclusions after comparative analysis are as follows:

Figure 12a shows that the anhydrite and quicklime ratio of 50:50 has a large number of flaky single sulfosalicate stacks and long needle-like AFt staggered crosses. Because of the large amount of quicklime, the hydration reaction of the slurry is rapid and consists of a large number of ettringite (AFt) crystals winding through the anhydrite and CSA clinker that have not had time to participate in the reaction, although the amount of anhydrite is not sufficient, and the ettringite (AFt) is not sufficiently stable to be converted into a large number of stacked alumina ferric-oxide (AFm) and other monosulfate crystals.

Figure 12b shows that the AFt skeletal structure contains a significant quantity of aluminum hydroxide (AH3) gel for an anhydrite and quicklime mixture ratio at 80:20 and needle-like ettringite (AFt) crystals appear in the form of nucleation points emitting multiple clusters ettringite (AFt) which contribute to the strength improvement to a certain extent and thus better mechanical properties (Figure 5).

As shown in Figure 12c, when the anhydrite dosage was 10%, the formation of pin-rods of ettringite (AFt) was observed, as well as a large number of flakes of alumina ferric-oxide (AFm), which was consistent with the physical phase detected in the XRD peaks. However, the hydration products were poorly developed and not tightly connected, as shown in the figure, so macroscopically, the mechanical properties were poorer at this low anhydrite dosage.

When the anhydrite is 80%, the XRD pattern of Figure 12d shows that the system is rich in ettringite (AFt) crystals and plume-shaped alumina hydroxide clusters, which exhibit higher strength, and a large number of radiating ettringite (AFt) crystal clusters that mechanically compact with each other to form the structure. The overall structure is denser and is filled inside the skeleton, thus forming a dense structure and increasing its hardness. In addition, the appearance of rod-shaped gypsum inside the calcium alumina skeleton structure, which is also detected in XRD (Figure 11), is due to the partial hydration of the remaining anhydrite to form gypsum, which is explained by the fact that when the concentrations of calcium ion ${(Ca}^{2+})\;$and sulfate ion $\left({SO}_{4}^{2-}\right)\;$in the solution exceeds the supersaturation of gypsum, the calcium ion ${(Ca}^{2+})\;$and sulfate ion $\left({SO}_{4}^{2-}\right)$ in the liquid phase becomes more concentrated. They and water molecules will spontaneously combine to form gypsum and precipitate crystals, which enhances the strength.

When anhydrite dosage is 100%, the XRD pattern of Figure 12e shows that there is a large amount of well-developed columnar ettringite (AFt) generated that is not involved in the reaction of anhydrite, as in the local enlargement. The large number of cracks is one of the reasons for the strength being slightly reduced with increasing anhydrite content. Anhydrite at this dosage has the greatest effect on the expansion rate (Figure 8) of CSAGM and causes rapid generation of a large amount of calcium ettringite (AFt) in the slurry after solidification expansion and no new material generation, so it is easy to reduce its strength due to expansion.

## 4. Conclusions

An early-stren grouting material was developed using calcium sulfoaluminate cement clinker (CSA), anhydrite, and quicklime. To determine the influence of anhydrite on the mechanical performance of CSA cement-based grouting material, laboratory experiments were conducted (within 1 d, 14 d, and 28 d) to evaluate the change in the setting time, expansion rate, slurry pH, and compressive strength. The hydration mechanism and microstructure properties of the grouting material were analyzed using XRD and SEM. Based on these experimental procedures, the following conclusions were drawn:

(1) Due to the relatively fast dissolution rate of anhydrite, the high strong and ettringite formation can be completed before 3 h of hydration when anhydrite is selected at a ratio (≥40%), causing non-expansion characteristic on the specimen hardened body in the later stage of hydration. The optimal dose of anhydrite in the CSA grouting material paste is 80%. (80% when it is incorporated alone, and 80:20 when it is incorporated with quicklime). This dose increases the ultimate strength at all ages and makes up 42.5% and 38.5% of the compressive strength independent or dependent on quicklime.
(2) The incorporation of anhydrite alone does not affect the pH of the grouting material paste compared to anhydrite–quicklime dosage. It increases the pH value of the grouting material paste and releases a large amount of hydroxide (OH^−^), which increases the alkalinity of the system This significantly prolongs the setting time of the CSA grouting material paste but does not have a direct effect on the ettringite production, nor the early or final strength.
(3) The equilibrium increase in the incorporation of the anhydrite–quicklime ratio slows the dissolution rate of the paste, leads to the expansion rate, and constrains the strength, thus leading to unified ettringite production. Increasing the anhydrite dosage inhibited the expansion performance and increased the strength. Besides, a uniformly distributed ettringite (AFt), calcium silicate hydrate, (C-S-H), and aluminum hydroxide (AH_3_) were produced.
(4) The incorporation of anhydrite has a positive significant effect on the dicalcium silicate (β-C_2_S) hydration products of CSAGM. The XRD and SEM characterizations confirmed that the hydration products of the CSAGM system are rich in needle-like AFt crystals, and plume-shaped aluminum hydroxide (AH3), which contribute to strength improvement. The hydration of dicalcium silicate (β-C_2_S) is inhibited by forming calcium silicate hydrate (C-S-H), due to its dissolution into calcium hydroxide, which produces trace amounts of katoite (C_2_ASH_8_) as the hydration product.

## Figures and Tables

**Table 1:** Mineralogical composition of the CSA clinker/wt.%.

Mineral	$C_{4}A_{3}\bar{S}$	β–C_2_S	C_2_F	Fe_2_O_3_
wt.%	58.64	24.43	6.41	1.92

**Table 2:** Chemical composition of the CSA clinker/wt.%.

Oxide	Fe_2_O_3_	TiO_2_	Al_2_O_3_	CaO	MgO	SiO_2_	SO_3_	LOI
wt.%	2.21	1.45	29.54	45.36	1.94	10.59	8.45	0.46

LOI = loss on ignition.

**Table 3:** Chemical composition of the anhydrite/wt.%.

Oxide	SiO_2_	CaO	Al_2_O_3_	MgO	Fe_2_O_3_	SO_3_	TiO_2_	Loss
wt.%	2.83	39.5	0.23	1.36	0.03	54.16	1.17	0.75

**Table 4:** The influence of anhydrite content on the CSAGM.

Sample Number	1	2	3	4	5	6	7	8	9	10	11
CSA	1	1	1	1	1	1	1	1	1	1	1
anhydrite	0	10%	20%	30%	40%	50%	60%	70%	80%	90%	100%

Note: The sample content of CSA cement and anhydrite in Table 4 is changed from 10:1 to 1:1, and their ratio does not necessarily depend on each other’s content to summarize the composition.

**Table 5:** The influence of anhydrite + quicklime ratio on the CSAGM.

Sample Number	Slurry A	Slurry B	
	CSA	Anhydrite	Quicklime
1-A/B(50:50)	100 wt.%	50 wt.%	50 wt.%
2-A/B(75:25)	100 wt.%	75 wt.%	25 wt.%
3-A/B(80:20)	100 wt.%	80 wt.%	20 wt.%
4-A/B(85:15)	100 wt.%	85 wt.%	15 wt.%

## Data Availability

The data presented in this study are available on request from the corresponding author due to privacy.

## Abbreviations

The following abbreviations are used in this manuscript:

CSA	Calcium sulfoaluminate
CSAGM	Calcium sulfoaluminate grouting mater
wt.%	Weight percentage
pH	Potential of hydrogen
XRD	X-ray diffraction
SEM	Scanning electron microscopy
E_X_	Expansion rate
L_X_	Length (test block)
L_1_	Initial length (test block)
